# Disordered pancreatic and gastrointestinal hormone responses to nutrient stimulation in Zambian children with severe acute malnutrition

**DOI:** 10.1113/EP093962

**Published:** 2026-08-15

**Authors:** Ellen Besa, Ruth Phiri, Andreck Tembo, Gwen Nayame, Amon Ngongola, Jens Rehfeld, Bolette Hartmann, Gary Frost, Douglas C. Heimburger, Paul Kelly

**Affiliations:** ^1^ Tropical Gastroenterology and Nutrition Group School of Medicine University of Zambia Lusaka Zambia; ^2^ Children's Hospital University Teaching Hospitals Lusaka Zambia; ^3^ Department of Clinical Biochemistry Rigshospitalet Copenhagen University Copenhagen Denmark; ^4^ Department of Metabolism Digestion and Reproduction Imperial College London London UK; ^5^ Vanderbilt Institute for Global Health Vanderbilt University Medical Center Nashville Tennessee USA; ^6^ Blizard Institute Centre for Immunobiology Barts and the London School of Medicine and Dentistry Queen Mary University of London London UK

**Keywords:** Africa, glucagon‐like peptides, insulin, nutrient sensing

## Abstract

Nutrient sensing enables the body to detect and respond to nutrient availability through hormonal regulation. In this study, we examined nutrient‐stimulated hormonal responses in children with severe acute malnutrition (SAM) compared with hospitalized (HC) and community (CC) child comparators. Basal and fed blood samples were collected from participants before and after consumption of a liquid meal. In the basal state, children with SAM showed significantly higher circulating concentrations of insulin, C‐peptide, peptide tyrosine tyrosine (PYY), ghrelin, glucagon, glucagon‐like peptide 1 (GLP‐1) and glucagon like peptide 2 (GLP‐2), in comparison to one or both comparators. After feeding, insulin, C‐peptide, cholecystokinin, PYY and GLP‐1 increased in all groups, indicating some level of preserved nutrient sensing. GLP‐2 increased significantly in SAM and hospitalized children, and a reduction in ghrelin was observed in children with SAM. No significant changes were detected for glucagon, leptin or secretin in any group. Disordered responses, defined as zero or positive postprandial responses in ghrelin and zero or negative postprandial responses in the other hormones, were seen in all children. Significantly higher percentages of children with SAM had disordered responses compared with the non‐malnourished children, particularly for insulin (23%) and C‐peptide (16%), suggestive of altered pancreatic hormonal regulation in SAM. These findings highlight the need for improved nutritional interventions for affected children.

## Abstract (highlights)

- **What is the central question of this study?**
  We set out to test the hypothesis that children with severe acute malnutrition have disordered hormonal responses to feeding in comparison to non‐malnourished children.
- **What is the main finding and its importance?**
  Children with severe acute malnutrition show elevated basal levels of insulin, C‐peptide, glucagon, peptide tyrosine tyrosine and glucagon‐like peptides 1 and 2, in addition to disordered insulin and C‐peptide post‐feeding responses. This indicates impaired pancreatic function and hormonal dysregulation, which might reduce the effectiveness of standard feeding therapies. The findings highlight the need for targeted nutritional interventions to improve metabolic recovery in affected children.

## 1 INTRODUCTION

Nutrient sensing (the process by which the body detects and responds to the availability of nutrients) is essential for growth, development and metabolic regulation across all organisms (Lee et al., 2025). It plays a fundamental role in cellular growth, proliferation, metabolic adaptation and disease prevention (Sung et al., 2023). In the gastrointestinal tract, specialized enteroendocrine cells detect nutrients via specific receptors (Lu et al., 2021). Once ligand–receptor binding has occurred, various nutrient‐sensing pathways are triggered, resulting in release of >20 different gut hormones crucial for maintaining metabolic homeostasis (Davison et al., 2025; Duca et al., 2021). In addition to the gastrointestinal tract, the pancreas also serves a crucial endocrine role by producing hormones that regulate food intake and energy balance (Kaprinska, 2022).

Undernutrition arises from a mismatch between nutrient or energy intake and the physiological demands of the body for growth and maintenance. It has been hypothesised that in response to undernutrition, children adapt metabolically to preserve energy and selectively protect vital organs and tissues (Page et al., 2025). Severe acute malnutrition (SAM; defined by a weight‐for‐height/length *z*‐score below −3 SD, mid upper arm circumference < 115 mm and/or the presence of bilateral pitting oedema) is associated with long‐term physiological consequences and contributes to an estimated 45% of deaths among children <5 years of age (Alflah & Alrashidi, 2023; Madewell et al., 2024). Nutritional supplementation is a key component of SAM management (WHO guidelines, 2023); however, in cases of complicated SAM, the response to therapeutic feeding remains suboptimal. This limited impact has been attributed, in part, to disruptions in intestinal barrier function, impaired nutrient absorption and microbial translocation, which are features commonly linked to enteropathy and concurrent infections (Keats et al., 2021). In Zambia, children with SAM have been shown to exhibit enteropathy characterized by epithelial abnormalities in the small intestine, including micro‐erosions, defective cell–cell adhesion and impaired function of secretory cells, all of which can compromise nutrient absorption (Amadi et al., 2017; Mulenga et al., 2022).

Previous studies have examined alterations in hormone levels in children with SAM, particularly in relationship to treatment response and mortality risk (Bartz et al., 2014; Francis, 2017; Freemark, 2015; Kilic et al., 2004; Martins et al., 2011; Prentice et al., 2002; Soliman et al., 2000), but there remains a paucity of data on the acute hormonal response to nutrient intake in the context of SAM and environmental enteropathy. Studying these responses is important because it can help to identify underlying mechanisms contributing to SAM and can provide insights into the impact of environmental enteropathy on nutrient absorption and utilization. The aim of this study was to assess the impact of nutrient stimulation on the release of circulating pancreatic and gastrointestinal hormones in children with malnutrition‐associated enteropathy.

## 2 MATERIALS AND METHODS

### 2.1 Ethical approval

Written informed consent was obtained from parents/guardians of children who were recruited into this study, and ethical approval to conduct the study was obtained from the University of Zambia Biomedical Ethics Committee (UNZABREC reference numbers 951‐2020 dated 14 July 2020 and 2025‐2021 dated 1 November 2021) and the National Health Research Authority (NHREB00004/02/12/2021 dated 6 December 2021). The study conformed to the standards set by the latest revision of the *Declaration of Helsinki* except for registration in a database.

### 2.2 Study design and participants

This study was a quasi‐experimental study to investigate the gut hormonal responses to nutrient stimulation in children with SAM and comparator groups. Three groups of children, one with SAM and two comparator groups (hospitalized and community children), were studied between June 2020 and July 2022 in the University Teaching Hospitals and the St. Augustine Clinic in Lusaka, Zambia. Eligibility for participation was determined prior to recruitment. After informed consent was obtained, a baseline questionnaire was administered to collect demographic information.

All children recruited into the study were between 6 and 60 months of age, whose parents were willing to consent to their participation in the study. Those with SAM (*n* = 43) were required to have a weight‐for‐height *z*‐score below −3, mid‐upper arm circumference < 115 mm and/or bilateral nutritional oedema on admission to hospital. These children were all admitted to the malnutrition ward of the Lusaka Children's Hospital, University Teaching Hospitals (UTH). The hospitalized children (HC; *n* = 26) were admitted to the paediatric surgical ward of the Lusaka Children's Hospital, UTH for elective surgery with a weight for height *z*‐score of >−3, and the community children (CC; *n* = 25) had to be resident in Misisi (a highly impoverished community in Lusaka, Zambia), have no reported illness at the time of recruitment and have WHZ scores of >−3.

### 2.3 Anthropometric measurements

Weight was measured using a mother–child scale (SECA 874, Hamburg Germany), length using an infantometer (SECA 416, Hamburg Germany) and height using a UNICEF height board (Pimolchaisuksakorn Co. Ltd, Nonthaburi, Thailand). Wasting, stunting and underweight were assessed using weight for height/length (WHZ), height/length for age (HAZ) and weight for age *z*‐score calculated using the WHO Anthropometry software v.3.2.2.

### 2.4 Food administration

Children were provided with a nutritionally similar 100 kcal liquid meal of either Pediasure (HC) or F100 (SAM and CC). The SAM children were given an appetite test before being transitioned from F75 to F100, and those from the community were reported to be feeding well. All the hospitalized children (HC) consumed 150 mL of the liquid meal, and the children in the other two groups consumed the meal ad libitum, with a median volume of 120 mL [interquartile range (IQR) 100–190 mL] in the SAM children and median of 175 (IQR 125–200 mL) in the community children.

### 2.5 Blood sample collection

Blood samples (2 mL) were collected into EDTA tubes (Becton Dickinson, UK) containing 20 µL of DPP4 inhibitor (Merck Millipore, Germany) and 100 µL of Pefabloc (Roche Diagnostics, Germany). Basal samples were collected ≥2.5 h after the last meal, and fed samples were collected 30 min after consumption of a meal. Plasma was collected after centrifugation of the blood samples at 856 × g for 15 min and was aliquoted and stored at −80°C until analysis.

### 2.6 Hormone assays

Hormone assays were run using an active GLP‐2 ELISA kit (Merck Millipore, Germany) and a Milliplex Metabolic Hormone Panel (Merck Millipore, Germany) specific for C‐peptide, acylated ghrelin, glucagon, active GLP‐1, PYY, insulin, leptin and secretin. Where available, samples were also sent for cholecystokinin (CCK) RIA (Rehfeld JF, 1988) and for comparative GLP‐1 ELISA (Mercodia, Sweden) analysis to Rigshospitalet, University of Copenhagen, Denmark. Assay sensitivity and the intra‐ and interassay coefficients of variation (expressed as a percentage) are provided in Table 1.

**TABLE 1:** Assay sensitivity, intra‐ and interassay variations.

Hormone	Assay	Assay sensitivity	Intra‐assay coefficient of variation (%)	Interassay coefficient of variation (%)
GLP‐2 total	Millipore ELISA	0.3 ng/mL	3.89	3.19
GLP‐1	Mercodia ELISA	<0.65 pmol/L	<7	<12
GLP‐1 active	Millipore multiplex assay	2.9 pg/mL	2.03	4.45
C‐Peptide		17.0 pg/mL	1.68	2.03
Ghrelin acylated		15 pg/mL	1.29	5.52
Glucagon		10 pg/mL	2.03	3.74
Insulin		57 pg/mL	2.09	8.04
Leptin		52 pg/mL	O.97	NA^a^
PYY		21 pg/mL	6.44	4.43
Secretin		12 pg/mL	0.41	3.18
CCK	RIA	0.1 pmol/L	<8	<12

^a^ NA, not applicable (interassay variation cannot be provided because leptin results were available from only a single assay plate).

### 2.7 Statistical analysis

Data were entered into Excel and exported into STATA v.17 (College Station, TX, USA) for statistical analysis. GraphPad Prism (GraphPad Software, USA) was also used for analysis and presentation of graphs. All continuous variables were checked for normality using the Shapiro–Wilk test and for hypothesis testing using the Kruskal–Wallis test. The Wilcoxon matched pairs signed rank test was used to compare basal and fed circulating hormone values and the Wilcoxon rank‐sum test to determine whether hormone levels differed by sex. To quantify the individual changes in hormone levels, basal hormone levels were subtracted from the fed levels to obtain a change (delta) value, and the Kruskal–Wallis test was used to compare groups. Fisher's exact test was used to compare the different proportions of children with disordered responses across the groups. Given that there are no defined hormone reference ranges in the Zambian population, we took a conservative approach, in which we declared, for the purposes of this paper, zero or positive responses in ghrelin and zero or negative responses in the other hormones to be disordered. GLP‐1 concentrations in picograms per millilitre were converted to picomoles per litre using the method described by Watkins et al. (2023) to allow for comparison between multiplex and single assay GLP‐1 results. Correlation testing was done using Spearman's rank correlation. A *ρ*‐value of ≥0.3 and *P*‐value of <0.05 were considered to be indicative of moderate to strong correlation. Linear regression analysis was used to adjust for the effect of human immunodeficiency virus (HIV). Missing data are attributable to invalid results or insufficient sample volumes. Missing data were treated as missing, and no imputation was made.

## 3 RESULTS

### 3.1 Participant characteristics

In all three groups, there were more males recruited than females, and community controls were older than in the other two groups (Table 2). The high number of males in the SAM and hospitalised groups reflected the higher number of boys among malnourished children nationally (ZSA et al., 2024) and the elective surgery schedule, which included a significant number of circumcision procedures. Baseline characteristics are as shown in Table 1.

**TABLE 2:** Table of baseline demographic and nutritional measurements.

Parameter	Children with SAM (*n* = 43)	Hospitalized children (*n* = 26)	Community children (*n* = 25)	*P*‐value
Sex, male:female	29:14	24:2	14:11	0.01
Age, months, median (IQR)	20 (15, 26)	31 (20, 44)	44 (38, 51)	<0.01
Weight for age *z*‐score, median (IQR)	−3.91 (−4.51, −3.04)	−0.83 (−1.84, 0.02)	−0.6 (−1.08, 0.04)	<0.01
Height for age *z*‐score, median (IQR)	−3.64 (−4.35, −2.28)	−0.96 (−2.71, −0.02)	−1.46 (−2.08, −1)	<0.01
Weight for height *z*‐score, median (IQR)	−2.89 (−4.14, −1.78)	−0.47 (−1.55, 0.17)	0.59 (−0.42, 1.14)	<0.01
HIV status (R:NR)	7:36	0:26	0:25	<0.01

Abbreviations: HIV, human immunodeficiency virus; IQR, interquartile range; R:NR, HIV reactive:non‐reactive; SAM, severe acute malnutrition.

### 3.2 Basal pancreatic and gastrointestinal hormone levels

Basal blood samples were taken from all children after a minimum time of 150 min without food. The median time of basal sample collection after the last meal was 240 min for hospitalized children (IQR 240–360 min), 210 min for SAM children (IQR 150–240 min) and 203 min for community children (IQR 186–236 min). Children with SAM exhibited higher basal circulating concentrations of pancreatic (Figure 1a–c) and L‐cell (Figure 1d–f) hormones in comparison to at least one comparator group. The levels of glucagon and ghrelin in the SAM children did not differ significantly from those of hospitalized controls, and PYY levels in SAM children were similar to those in community children. No significant difference in basal concentrations was noted for secretin, leptin or CCK (Figure 1h–j). Significant differences were noted between the two comparator groups, in that hospitalized children had higher glucagon, GLP‐2 and ghrelin levels than community children (Table 3).

**FIGURE 1:**
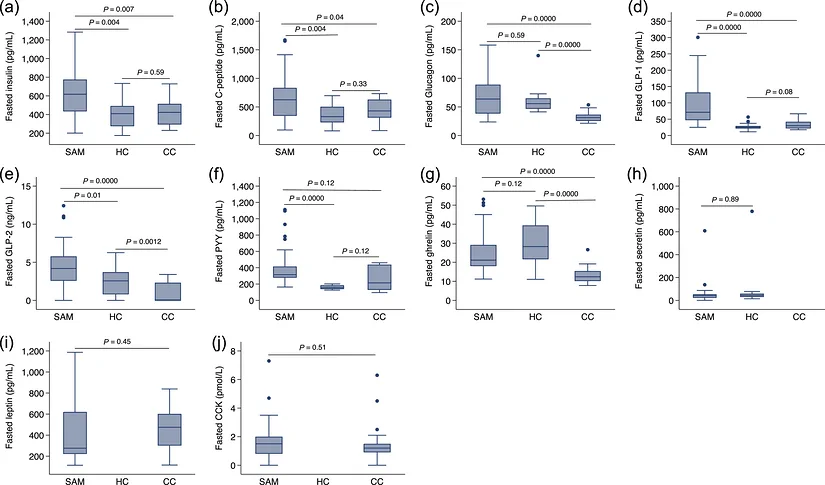
Basal circulating hormone levels across the groups. Boxplots display medians and interquartile ranges. Group sizes (*n*) for each analyte are as follows: insulin, C‐peptide and glucagon (SAM, *n* = 35; HC, *n* = 17; CC, *n* = 17); GLP‐1 (SAM, *n* = 35; HC, *n* = 16; CC, *n* = 17); GLP‐2 (SAM, *n* = 35; HC, *n* = 26; CC, *n* = 17); PYY and ghrelin (SAM, *n* = 35; HC, *n* = 16; CC, *n* = 16); secretin (SAM, *n* = 24; HC, *n* = 16); leptin (SAM, *n* = 16; CC, *n* = 16); and CCK (SAM, *n* = 25; CC, *n* = 23). Differences between groups were calculated using the Kruskal–Wallis test, and *P*‐values are shown in each panel. Abbreviations: CC, community children; HC, hospitalised children; SAM, severe acute malnutrition.

**TABLE 3:** Basal and postprandial hormone levels.

	Children with SAM			Hospitalized children			Community children		
Hormone	Basal	Fed	*n*	Basal	Fed	*n*	Basal	Fed	*n*
Insulin, pg/mL	617 (434–775)	674 (473–986)	35	409 (283–492)	648 (461–725)	17	423 (293–514)	907 (699–1696)	17
C‐Peptide, pg/mL	625 (344–834)	1115 (710–1569)	35	330 (233–503)	969 (602–1530)	17	430 (312–629)	1284 (1021–2015)	17
Glucagon, pg/mL	63.8 (38.4–89.0)	71.1 (45.1–92.6)	35	55.7 (46.7–65.1)	56.7 (51.5–71.2)	17	31.3 (25.5–36.5)	36.7 (27.2–42.7)	17
GLP‐1, pg/mL	71.1 (47.0–132)	118 (77.1–162)	35	24.3 (19.7–29.3)	47.1 (37.4–59.0)	16	29.9 (21.6–42.1)	56.4 (37.7–64.3)	17
GLP‐2, ng/mL	4.19 (2.56–5.78)	5.03 (3.82–6.84)	35	2.55 (1.59–3.67)	3.67 (3.07–4.93)	26	0 (0–2.32)	1.74 (0–3.02)	17
PYY, pg/mL	315 (278–415)	360 (288–521)	35	155 (137–183)	192 (163–214)	16	267 (125–439)	292 (189–435)	16
Ghrelin, pg/mL	21.1 (18.0–29.1)	21.1 (14.7–30.3)	35	28.2 (23.5–36.6)	27.0 (22.7–29.1)	16	12.4 (10.2–15.4)	10.7 (8.8–15.5)	16
Secretin, pg/mL	44.6 (30.1–56.4)	40.5 (28.6–55.0)	24	38.3 (28.3–54.5)	42.5 (32.8–56.0)	16	–	–	–
Leptin, pg/mL	276 (221–620)	232 (160–555)	16	–	–		455 (263–587)	430 (297–583)	16
CCK, pmol/L	1.50 (0.80–2.00)	3.50 (2.10–5.50)	25	–	–		1.20 (0.90–1.50)	2.60 (1.70–4.90)	23

*Note*: Circulating gut hormone levels are expressed as the median and interquartile range (IQR).

Abbreviations: CCK, cholecystokinin; GLP‐1, glucagon‐like peptide 1; GLP‐2, glucagon‐like peptide 2; PYY, peptide tyrosine tyrosine; SAM, severe acute malnutrition.

### 3.3 Hormonal responses to feeding

After feeding, the pancreatic hormones insulin and C‐peptide showed a significant increase in all three groups, whereas no change was seen in glucagon (Figure 2). Significant increases in GLP‐1, CCK and PYY were observed in all three groups, while GLP‐2 concentrations increased in the SAM and hospitalized children but not in the community children (Figure 3). Significant postprandial ghrelin reduction was seen only in children with SAM, whereas secretin and leptin remained unchanged in all groups (Figure 3). Children with SAM had disordered insulin and C‐peptide postprandial responses (Table 4).

**FIGURE 2:**
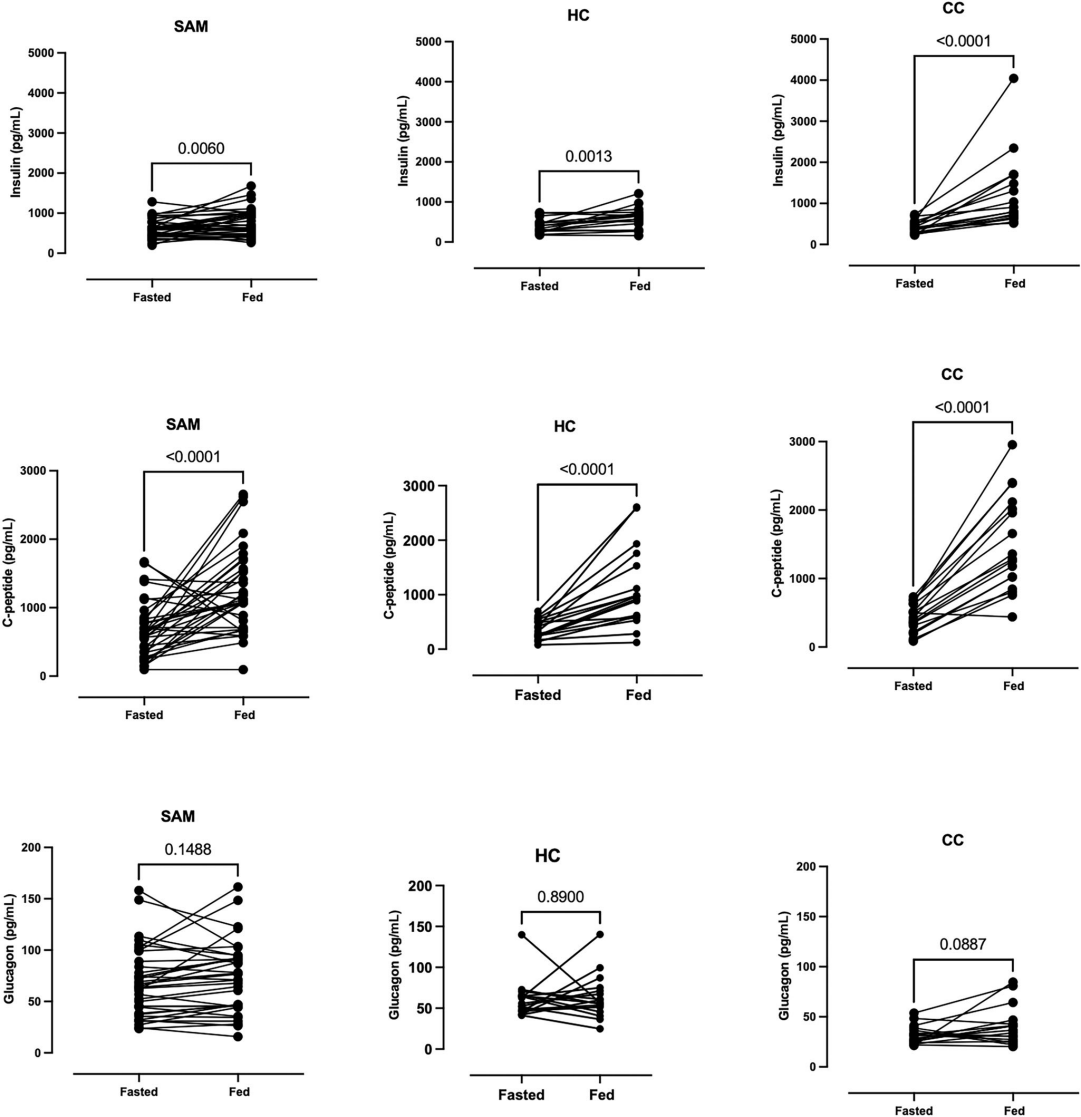
Pre‐ and postprandial pancreatic hormone levels in children with severe acute malnutrition, hospital controls and community controls. Parallel line plot showing paired data points for each individual. Abbreviations: CC, community children; HC, hospitalised children; SAM, severe acute malnutrition.

FIGURE 3Paired pre‐ and postprandial comparisons of gastrointestinal hormones in children with severe acute malnutrition, hospitalized children and community children. Abbreviations: CC, community children; CCK, cholecystokinin; GLP‐1, glucagon‐like peptide 1; GLP‐2, glucagon‐like peptide 2; HC, hospitalized children; PYY, peptide tyrosine tyrosine; SAM, severe acute malnutrition.

**Figure:**
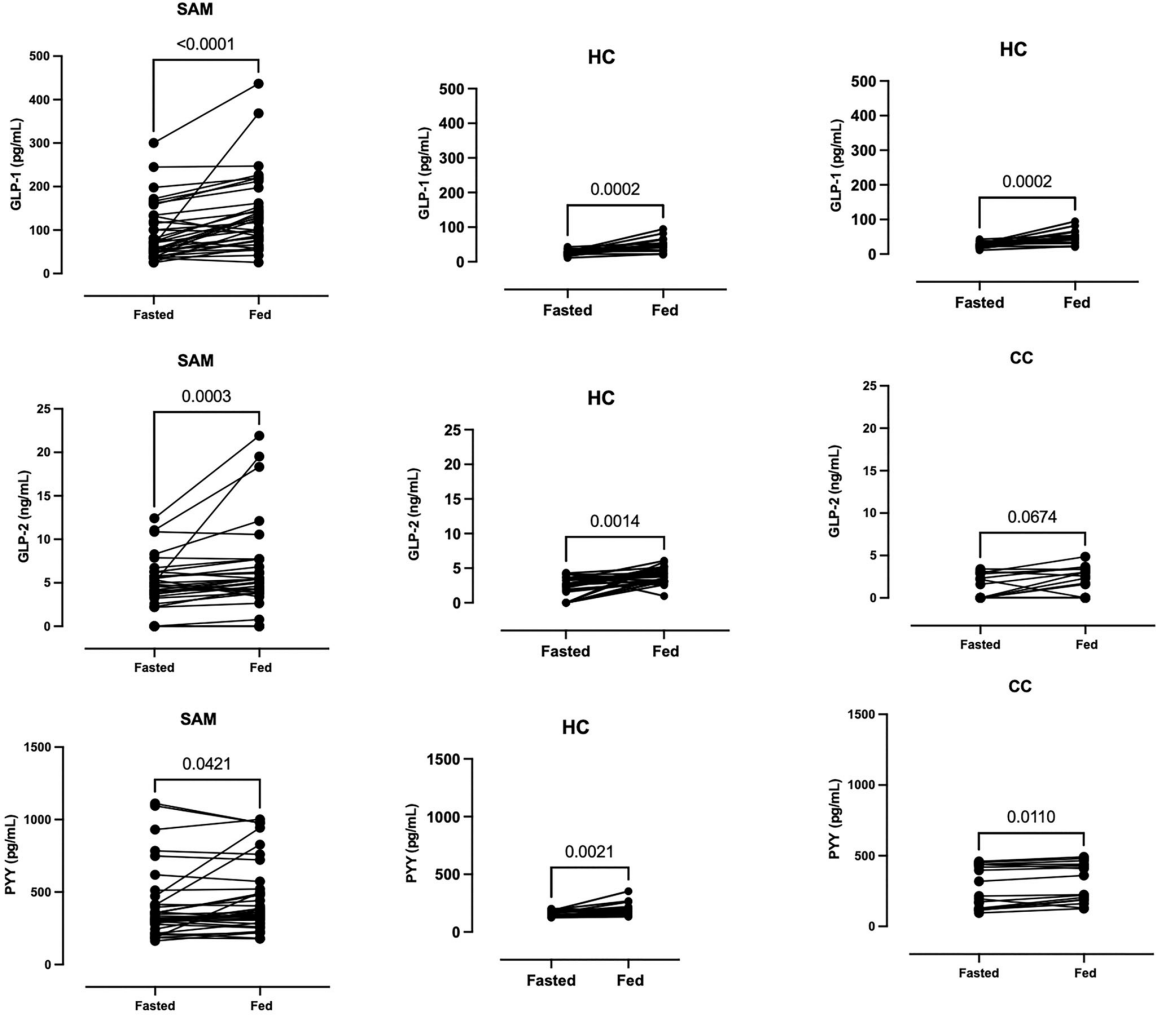

**Figure:**
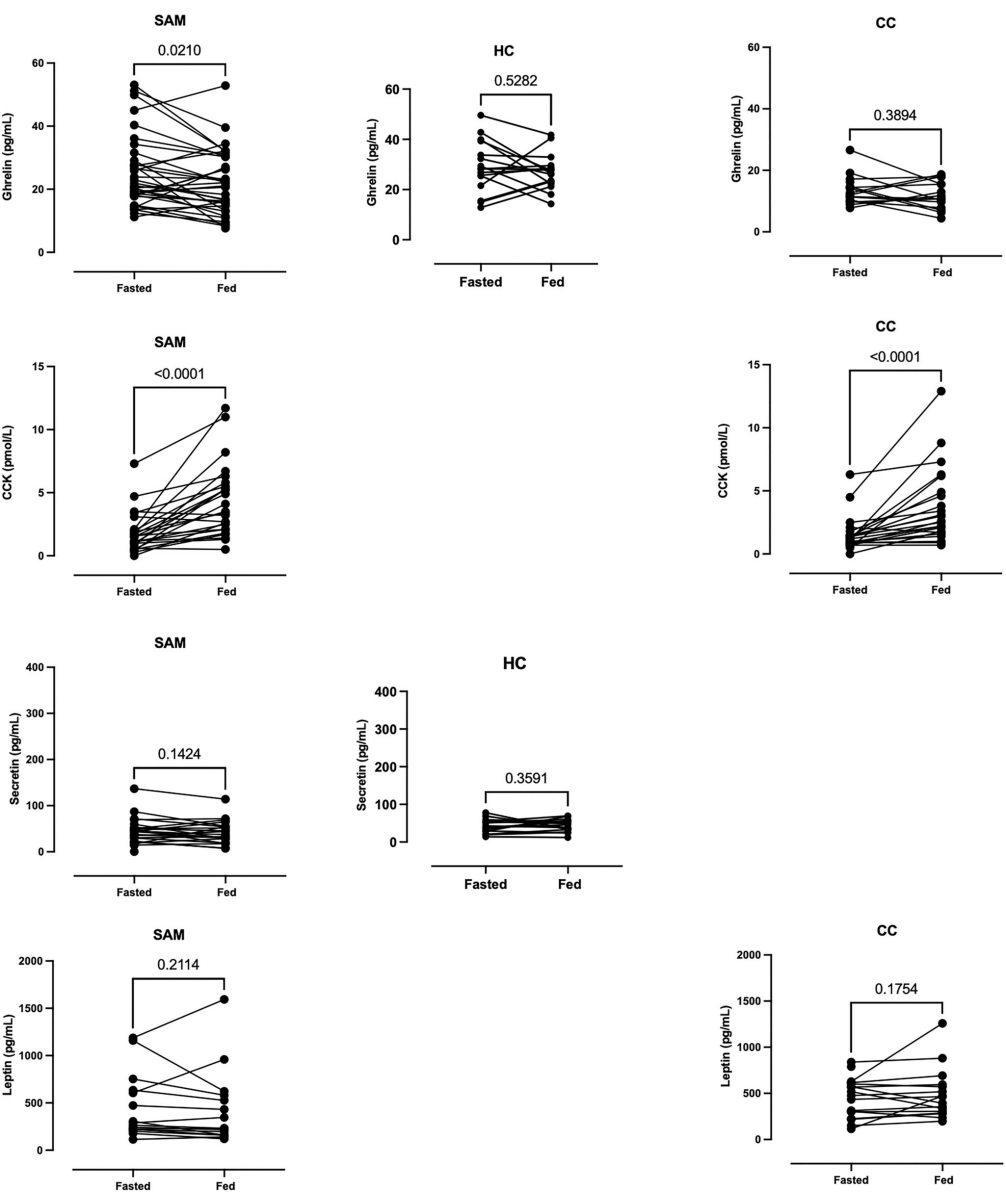

**TABLE 4:** Disordered hormone responses to a test meal.

	Children with SAM		Hospitalized children		Community children		
Hormone	Disordered responses (*n*)	Percentage	Disordered responses (*n*)	Percentage	Disordered responses (*n*)	Percentage	*P*‐value
Ghrelin	11/43	25.6	7/26	26.9	7/25	28	1
Leptin	11/16	68.8	–	–	4/16	25	0.03
Cholecystokinin	3/43	6.98	–	–	3/25	12	0.66
Secretin	14/43	32.6	8/26	30.8	–	–	0.55
Insulin	10/43	23.3	3/26	11.5	0/25	0	0.02
C‐peptide	7/43	16	0/26	0	1/25	4	0.04
Glucagon	14/43	32.6	8/26	30.8	7/25	28	0.96
GLP‐1	5/43	11.7	2/26	7.7	0/25	0	0.30
GLP‐2	11/43	25	5/26	19.2	9/25	36	0.41
PYY	13/43	30.2	3/26	11.5	2/25	8	0.05

*Note*: Number and percentage of individuals with disordered hormone responses is defined as a delta value of zero or positive for ghrelin and zero or negative for all other hormones. Red values indicate significant *p* values (*p* < 0.05).

Abbreviations: GLP‐1, glucagon‐like peptide 1; GLP‐2, glucagon like peptide 2; PYY, peptide tyrosine tyrosine; SAM, severe acute malnutrition.

### 3.4 Correlation analysis

The WHZ score was correlated with insulin (*ρ* = 0.40, *P*< 0.05) and  leptin (*ρ* = 0.53, *P *< 0.05) delta values (Figure 4). Pairwise correlations of the basal and delta values showed correlations between the pancreatic hormones (glucagon, insulin and C‐peptide), CCK, ghrelin and L‐cell‐derived hormones (GLP‐1, GLP‐2 and PYY). The basal values showed stronger correlations than the delta values (Figure 5). Age was negatively correlated with circulating glucagon (basal, *ρ* = −0.57, *P* < 0.01; fed, *ρ* = −0.38, *P* < 0.01), GLP‐1 (basal, *ρ* = −0.40, *P* < 0.01; fed, *ρ* = −0.42, *P* < 0.01) and GLP‐2 (basal, *ρ* = −0.45, *P* < 0.01; fed, *ρ* = −0.55, *P* < 0.01) and ghrelin in the basal state only (*ρ* = −0.36, *P* < 0.01). HIV and sex had no bearing on circulating hormone levels. GLP‐1 ELISA and multiplex results conducted in two different laboratories (*ρ* = 0.87, *P* < 0.01) were also strongly correlated, especially at <30 pmol/L.

**FIGURE 4:**
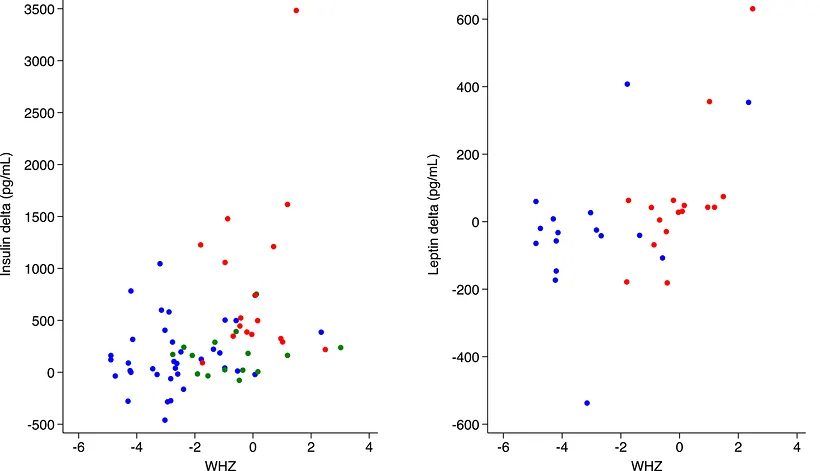
Scatter graphs of delta insulin (*ρ* = 0.40, *P *< 0.01) and leptin levels (*ρ* = 0.53, *P* < 0.01) with weight for height *z*‐score. Blue circles, children with severe acute malnutrition; green, hospitalized children; and red, community children. Abbreviation: WHZ, weight for height *z*‐score.

**FIGURE 5:**
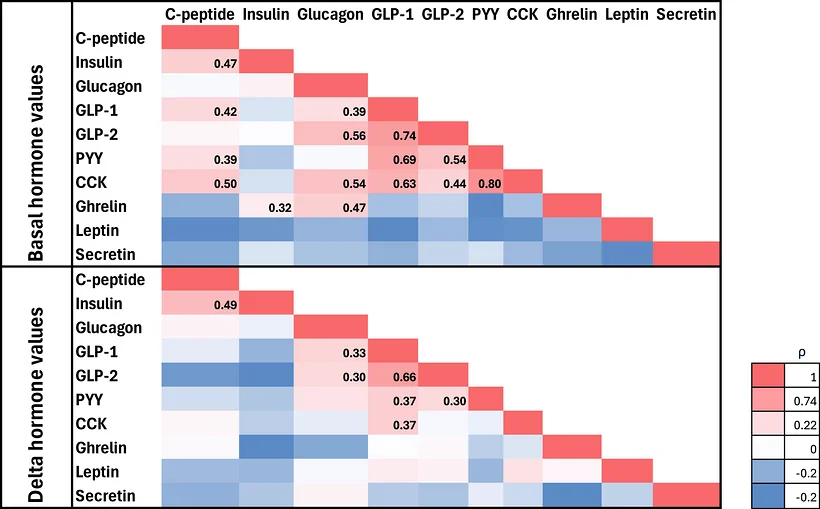
Heat map of correlation coefficients derived from pairwise correlations of basal (a) and delta (b) hormone values. The numbers shown in the boxes are correlation coefficients (*ρ*) for statistically significant correlations with *P* < 0.05. Abbreviations: CCK, cholecystokinin; GLP‐1, glucagon‐like peptide 1; GLP‐2, glucagon‐like peptide 2; PYY, peptide tyrosine tyrosine.

## 4 DISCUSSION

Nutritional rehabilitation remains a cornerstone of treatment for children with SAM. However, evidence suggests that anthropometric recovery does not necessarily indicate full metabolic or physiological restoration, with children who have experienced SAM being more vulnerable to long‐term metabolic disruptions (Di Giovanni et al., 2016) and poor health outcomes (Bwakura‐Dangarembizi et al., 2022; CHAIN Network, 2022). It has been hypothesised that circulating gut hormone responses might be delayed or dysregulated owing to persistent intestinal mucosal damage or altered gut microbiota (Martin et al., 2019; Mulenga et al., 2022; Smith et al., 2013). In this study, we aimed to evaluate whether children with SAM and enteropathy could mount appropriate acute hormonal responses to a 100 kCal test meal.

In the basal state, children with SAM, when compared with the other groups, showed higher levels of all measured hormones except ghrelin, leptin, secretin and CCK. Higher basal hormone levels in those with SAM are likely to be attributable to a complex interplay of intestinal, metabolic and compensatory mechanisms. Basal insulin levels in the comparator groups were similar to what has been reported in fasted children with normal weight for height *z*‐scores (Francis, 2017), whereas the SAM children had high insulin levels comparable to levels seen in obese prepubertal children (Lomenick et al., 2009), which might be evidence of insulin resistance. A study of hormonal status in children with SAM measured fasted hormones at three intervals (enrolment; after 2 weeks of inpatient care or at time of discharge from inpatient care; and after 4–10 weeks of outpatient treatment) and found that insulin levels were reduced at enrolment, with a 50% increase at the 2 week time point (Bartz et al., 2014). Although it has been shown that insulin levels return to preprandial levels within 2–3 h (Kapur et al., 2010), the insulin levels reported in our study were higher than has been reported in children with SAM, and we are uncertain whether this is attributable to prior feeding, stimulation by other gastrointestinal hormones or pancreatic dysfunction. Several studies have shown reduced leptin and insulin and increased PYY and GLP‐1 in fasted children with SAM (Akib et al., 2021 **;** Deng et al., 2024; Freemark, 2015; Haspolat et al., 2007; Palacio et al., 2002). The increased GLP‐1/PYY observed in malnourished children might contribute to appetite suppression, resulting in anorexia often seen in SAM children (Page et al., 2025), and the increased GLP‐2 might be indicative of a heightened response to gut damage and inflammation (Sturgeon et al., 2023). Although children with SAM had ghrelin levels that were higher than those of the community children but lower than the hospitalized children, the reported levels of ghrelin across the groups were far lower than has been seen in other studies of infants (De Fluiter et al., 2021; Kuppens et al., 2015 **;** Kweh et al., 2015). Low ghrelin levels, such as those seen in our study, have been reported in pre pubertal and pubertal paediatric subjects (Prodam et al., 2014) and in critically ill children (Martinez et al., 2020). The difference in basal ghrelin levels between the hospitalized children and the other two groups could possibly be explained by longer fasting times, because this was justified in those scheduled for surgical procedures. It is difficult to justify prolonged fasting in children without cause.

In response to nutrient stimulation, all groups had a marked increase in insulin, C‐peptide, GLP‐1, GLP‐2, PYY and CCK, but not in glucagon, leptin or secretin. Although insulin and C‐peptide are produced in equal amounts (Vinay et al., 2026), larger prandial changes were noted in C‐peptide compared with insulin. This is likely to be explained by the longer plasma half‐life and more stable concentrations of C‐peptide (Lin et al., 2025). A higher proportion of children with SAM exhibited disordered responses in insulin, C‐peptide and leptin. A study conducted in Gambia that looked at acute nutrient‐stimulated hormone responses in children with SAM (Nabwera et al., 2017) reported no differences in leptin and ghrelin levels after feeding or between cases and controls. Postprandial increase in leptin 2 h after a meal have been reported in lean subjects, with a reduction noted in obese subjects and no significant change in anorexic subjects (Korek et al., 2013). The disordered leptin response seen in our study population might be attributable to the time of sample collection, because most increases in serum leptin have been reported to occur 4–7 h after meals (Fried et al., 2000) and are small and variable (Korbonits et al., 1997). Acylated ghrelin responses to feeding vary, with no change noted in normal lean infants after a standard meal and with a decrease in prepubertal and pubertal paediatric subjects after an oral glucose tolerance test (Prodam et al., 2014). The observation that ghrelin levels in neither comparator group decreased after their respective meals suggests that their nutrition status rather than the carbohydrate content of the liquid feed might be driving this refactoriness. The F100 carbohydrate composition is similar to that of the oral glucose tolerance test, whereas the pediasure drink contains less carbohydrate. The reduction of ghrelin levels in SAM children after feeding could be indicative of suppressed appetite. Overall, the postprandial responses in the majority of hormones suggest some preservation of nutrient sensing irrespective of malnutrition status.

Interpretation of most hormone concentrations is limited by a lack of comprehensive reference values, because hormones can be dependent on sex, weight and age. A recent publication by Brandt‐Heunemann et al. (2026) has generated the first reported reference curves for leptin concentrations across the entire age and weight range using pooled European data, but these curves might not be applicable to African populations, because the reported median values in these Euopean children [females, median 1.6 ng/mL (IQR 1.0, 2.7ng/mL); and males, median 1.2 ng/mL (IQR 0.8, 2.1ng/mL)] are much higher than values reported in African children [median 0.18 ng/mL,(IQR 0.088, 0.343ng/mL); Deng et al., 2024]. Leptin has been associated with mortality in children with SAM (Bartz et al., 2014) and linked to improved nutrition status (Palacio et al., 2002). The strong correlations seen between hormones reinforce previous reports (Cho & Lim, 2024; Wortha et al., 2021).

Although the high basal hormone levels are suggestive of adaptive or compensatory mechanisms at play, coupled with preserved nutrient sensing as evidenced by fed hormone responses, it remains unclear whether these responses are sufficient to support full nutritional recovery in SAM.

A major limitation of this study was the inability to take repeated blood samples, but this was not considered feasible in this vulnerable population owing to ethical considerations. It is known that some gut hormones have biphasic secretion patterns. There is a clear need for further mechanistic studies to investigate gut hormone dynamics in children with malnutrition and their roles in recovery. The high prevalence of disordered hormone responses in children with SAM suggests potential benefit from therapeutic interventions, such as hormone analogues. Agents such as teduglutide, a GLP‐2 analogue, have shown promise in supporting intestinal adaptation and repair, and recent evidence from Zambian children suggests that it might be beneficial in this context (Chandwe et al., 2024).

## AUTHOR CONTRIBUTIONS

Study concept and design: Ellen Besa and Paul Kelly. Acquisition, analysis and interpretation of data: Ellen Besa, Ruth Phiri, Andreck Tembo, Jens Rehfeld, Bolette Hartmann, Paul Kelly, Amon Ngongola, Douglas C Heimburger. Statistical analysis: Ellen Besa and Paul Kelly. Obtained funding: Ellen Besa, Douglas C Heimburger, Paul Kelly. Initial drafting of the manuscript: Ellen Besa and Paul Kelly. Critical revision of the manuscript was done by all the authors. All authors had access to the study data and approved and reviewed the final manuscript. All authors agree to be accountable for all aspects of the work in ensuring that questions related to the accuracy or integrity of any part of the work are appropriately investigated and resolved, and all persons designated as authors qualify for authorship and all those who qualify are listed.

## CONFLICT OF INTEREST

None declared.

## GENERATIVE AI STATEMENT

The authors declare that no generative AI tools were utilised in the creation or editing of this manuscript.

## Data Availability

Data described in the manuscript, code book and analytic code will be made available on request pending approval.

## References

[eph70431-bib-0001] Akib, R. D. , Aminuddin, A. , Hamid, F. , Prihantono, P. , Bahar, B. , & Hadju, V. (2021). Leptin levels in children with malnutrition. Gaceta Sanitaria, 35, S278–S280. 10.1016/j.gaceta.2021.10.033 34929831

[eph70431-bib-0002] Alflah, Y. M. , & Alrashidi, M. A. (2023). Severe acute malnutrition and its consequences among malnourished children. Journal of Clinical Pediatric Research, 2(1), 1–5.

[eph70431-bib-0003] Amadi, B. , Besa, E. , Zyambo, K. , Kaonga, P. , Louis‐Auguste, J. , Chandwe, K. , Tarr, P. I. , Denno, D. M. , Nataro, J. P. , Faubion, W. , Sailer, A. , Yeruva, S. , Brantner, T. , Murray, J. , Prendergast, A. J. , Turner, J. R. , & Kelly, P. (2017). Impaired barrier function and autoantibody generation in malnutrition enteropathy in Zambia. EBioMedicine, 22, 191–199. 10.1016/j.ebiom.2017.07.017 PMC5552244 28750860

[eph70431-bib-0004] Bartz, S. , Mody, A. , Hornik, C. , Bain, J. , Muehlbauer, M. , Kiyimba, T. , Kiboneka, E. , Stevens, R. , Bartlett, J. , St Peter, J. V. , Newgard, C. B. , & Freemark, M. (2014). Severe acute malnutrition in childhood: Hormonal and metabolic status at presentation, response to treatment, and predictors of mortality. The Journal of Clinical Endocrinology and Metabolism, 99(6), 2128–2137. 10.1210/jc.2013-4018 PMC4037734 24606092

[eph70431-bib-0005] Brandt‐Heunemann, S. , Vogel, M. , Kiess, W. , Körner, A. , Blüher, M. , Meigen, C. , Stein, R. , Wenzel, E. , Landgraf, K. , von Schnurbein, J. , Denzer, C. , Lennerz, B. S. , Elad, L. , Kornmann, M. , Pridzun, L. , Rothenbacher, D. , Steinacker, J. M. , Janda, A. š. , Rutters, F. , … Wabitsch, M. (2026). Reference values for serum leptin levels in children, adolescents, and adults with normal weight, overweight, and obesity. The Journal of Clinical Endocrinology and Metabolism, 111(3), 832–844. 10.1210/clinem/dgaf439 40852975

[eph70431-bib-0006] Bwakura‐Dangarembizi, M. , Dumbura, C. , Amadi, B. , Chasekwa, B. , Ngosa, D. , Majo, F. D. , Sturgeon, J. P. , Chandwe, K. , Kapoma, C. , Bourke, C. D. , Robertson, R. C. , Nathoo, K. J. , Ntozini, R. , Norris, S. A. , Kelly, P. , & Prendergast, A. J. (2022). Recovery of children following hospitalization for complicated severe acute malnutrition. Maternal & Child Nutrition, 18, e13302. 10.1111/mcn.13302 PMC8932709 34939325

[eph70431-bib-0051] Chandwe, K. , Bwakura‐Dangarembizi, M. , Amadi, B. , Tawodzera, G. , Ngosa, D. , Dzikiti, A. , Chulu, N. , Makuyana, R. , Zyambo, K. , Mutasa, K. , Mulenga, C. , Besa, E. , Sturgeon, J. P. , Mudzingwa, S. , Simunyola, B. , Kazhila, L. , Zyambo, M. , Sonkwe, H. , Mutasa, B. , … Kelly, P. (2024). Malnutrition enteropathy in Zambian and Zimbabwean children with severe acute malnutrition: A multi‐arm randomized phase II trial. Nature Communications, 15(1), 2910. 10.1038/s41467-024-45528-0 PMC11024201 38632262

[eph70431-bib-0007] Cho, H. , & Lim, J. (2024). The emerging role of gut hormones. Molecules and Cells, 47(11), 100126. 10.1016/j.mocell.2024.100126 PMC11577206 39426686

[eph70431-bib-0008] Davison, A. , Reimann, F. , & Gribble, F. M. (2025). Molecular mechanisms of stimulus detection and secretion in enteroendocrine cells. Current Opinion in Neurobiology, 92, 103045. 10.1016/j.conb.2025.103045 40378579

[eph70431-bib-0009] De Fluiter, K. S. , Kerkhof, G. F. , van Beijsterveldt, I. A. L. P. , Breij, L. M. , van Vark‐van der Zee, L. C. , Mulder, M. T. , Abrahamse‐Berkeveld, M. , & Hokken‐Koelega, A. C. S. (2021). Appetite‐regulating hormone trajectories and relationships with fat mass development in term‐born infants during the first 6 months of life. European Journal of Nutrition, 60(7), 3717–3725. 10.1007/s00394-021-02533-z PMC8437841 33768316

[eph70431-bib-0010] Deng, L. , Argaw, A. , Guesdon, B. , Freemark, M. , Roberfroid, D. , Kemokai, I. A. , Mostak, M. R. , Alim, M. A. , Khan, M. A. H. , Muehlbauer, M. , Khan, M. S. T. , Bawo, L. , Dunbar, N. K. , Taylor, C. H. , Fouillet, H. , Huneau, J.‐F. , Lachat, C. , Kolsteren, P. , & Dailey‐Chwalibóg, T. (2024). Clinical and biochemical responses to treatment of uncomplicated severe acute malnutrition: A multicenter observational cohort from the OptiDiag study. The American Journal of Clinical Nutrition, 120(3), 570–582. 10.1016/j.ajcnut.2024.05.029 39232601

[eph70431-bib-0011] Di Giovanni, V. , Bourdon, C. , Wang, D. X. , Seshadri, S. , Senga, E. , Versloot, C. J. , Voskuijl, W. , Semba, R. D. , Trehan, I. , Moaddel, R. , Ordiz, M. I. , Zhang, L. , Parkinson, J. , Manary, M. J. , & Bandsma, R. H. (2016). Metabolomic changes in serum of children with different clinical diagnoses of malnutrition. Journal of Nutrition, 146(12), 2436–2444. 10.3945/jn.116.239145 PMC5118769 27807038

[eph70431-bib-0012] Duca, F. A. , Waise, T. M. Z. , Peppler, W. T. , & Lam, T. K. (2021). The metabolic impact of small intestinal nutrient sensing. Nature Communications, 12, 903. 10.1038/s41467-021-21235-y PMC7876101 33568676

[eph70431-bib-0013] Francis, N. K. (2017). Assessment of insulin sensitivity and its convalescence with dietary rehabilitation in undernourished rural West Bengal population. Journal of Clinical and Diagnostic Research, 11(5), LC29–LC32. 10.7860/JCDR/2017/25888.9937 PMC5483717 28658815

[eph70431-bib-0014] Freemark, M. (2015). Metabolomics in nutrition research: Biomarkers predicting mortality in children with severe acute malnutrition. Food Nutrition Bulletin, 36(1), S88–S92. 10.1177/15648265150361S114 25902620

[eph70431-bib-0015] Fried, S. K. , Ricci, M. R. , Russell, C. D. , & Laferrère, B. (2000). Regulation of leptin production in humans. The Journal of Nutrition, 130(12), 3127S–3131S. 10.1093/jn/130.12.3127S 11110887

[eph70431-bib-0016] Haspolat, K. , Ece, A. , Gürkan, F. , Atamer, Y. , Tutanç, M. , & Yolbaş, I. (2007). Relationships between leptin, insulin, IGF‐1 and IGFBP‐3 in children with energy malnutrition. Clinical Biochemistry, 40(3‐4), 201–205. 10.1016/j.clinbiochem.2006.11.008 17208213

[eph70431-bib-0017] Kapur, S. , Groves, M. N. , Zava, D. T. , & Kapur, S. (2010). Postprandial insulin and triglycerides after different breakfast meal challenges: Use of finger stick capillary dried blood spots to study postprandial dysmetabolism. Journal of Diabetes Science and Technology, 4(2), 236–243. 10.1177/193229681000400202 PMC2864157 20307382

[eph70431-bib-0018] Karpińska, M. , & Czauderna, M. (2022). Pancreas‐Its functions, disorders, and physiological impact on the Mammals' organism. Frontiers in Physiology, 13, 807632. 10.3389/fphys.2022.807632 PMC9005876 35431983

[eph70431-bib-0019] Keats, E. C. , Das, J. K. , Salam, R. A. , Lassi, Z. S. , Imdad, A. , Black, R. E. , & Bhutta, Z. A. (2021). Effective interventions to address maternal and child malnutrition: An update of the evidence. The Lancet Child & Adolescent Health, 5(5), 367–384. 10.1016/S2352-4642(20)30274-1 33691083

[eph70431-bib-0020] Kilic, M. , Taskin, E. , Ustundag, B. , & Aygun, A. D. (2004). The evaluation of serum leptin level and other hormonal parameters in children with severe malnutrition. Clinical Biochemisty, 37(5), 382–387. 10.1016/j.clinbiochem.2003.12.010 15087254

[eph70431-bib-0021] Korbonits, M. , Trainer, P. J. , Little, J. A. , Edwards, R. , Kopelman, P. G. , Besser, G. M. , Svec, F. , & Grossman, A. B. (1997). Leptin levels do not change acutely with food administration in normal or obese subjects, but are negatively correlated with pituitary‐adrenal activity. Clinical Endocrinology, 46(6), 751–757. 10.1046/j.1365-2265.1997.1820979.x 9274707

[eph70431-bib-0022] Korek, E. , Krauss, H. , Gibas‐Dorna, M. , Kupsz, J. , Piątek, M. , & Piątek, J. (2013). Fasting and postprandial levels of ghrelin, leptin and insulin in lean, obese and anorexic subjects. Gastroenterology Review, 8(6), 383–389. 10.5114/pg.2013.39922 PMC4027830 24868288

[eph70431-bib-0023] Kuppens, R. J. , Diène, G. , Bakker, N. E. , Molinas, C. , Faye, S. , Nicolino, M. , Bernoux, D. , Delhanty, P. J. , van der Lely, A. J. , Allas, S. , Julien, M. , Delale, T. , Tauber, M. , & Hokken‐Koelega, A. C. (2015). Elevated ratio of acylated to unacylated ghrelin in children and young adults with Prader‐Willi syndrome. Endocrine, 50(3), 633–642. 10.1007/s12020-015-0614-x PMC4662713 25989955

[eph70431-bib-0024] Kweh, F. A. , Miller, J. L. , Sulsona, C. R. , Wasserfall, C. , Atkinson, M. , Shuster, J. J. , Goldstone, A. P. , & Driscoll, D. J. (2015). Hyperghrelinemia in Prader‐Willi syndrome begins in early infancy long before the onset of hyperphagia. American Journal of Medical Genetics, 167(1), 69–79. 10.1002/ajmg.a.36810 PMC4391201 25355237

[eph70431-bib-0025] Lee, G. , Lee, J. , Suh, G. S. B. , & Oh, Y. (2025). Post ingestive systemic nutrient sensing for whole body homeostasis. Molecules and Cells, 48(11), 100271. 10.1016/j.mocell.2025.100271 PMC12495334 40886943

[eph70431-bib-0026] Lin, Y. , McCrimmon, R. J. , & Pearson, E. R. (2025). Exploring the potential role of C‐peptide in type 2 diabetes management. Diabetic Medicine, 42(3), e15469. 10.1111/dme.15469 PMC11823364 39797595

[eph70431-bib-0027] Lomenick, J. P. , Melguizo, M. S. , Mitchell, S. L. , Summar, M. L. , & Anderson, J. W. (2009). Effects of meals high in carbohydrate, protein, and fat on ghrelin and peptide YY secretion in prepubertal children. The Journal of Clinical Endocrinolog & Metabolism, 94(11), 4463–4471. 10.1210/jc.2009-0949 PMC2775646 19820013

[eph70431-bib-0028] Lu, V. B. , Gribble, F. M. , & Reimann, F. (2021). Nutrient‐Induced cellular mechanisms of gut hormone secretion. Nutrients, 13(3), 883. 10.3390/nu13030883 PMC8000029 33803183

[eph70431-bib-0029] Madewell, Z. J. , Keita, A. M. , Das, P. M.‐G. , Mehta, A. , Akelo, V. , Oluoch, O. B. , Omore, R. , Onyango, D. , Sagam, C. K. , Cain, C. J. O. , Chukwuegbo, C. , Kaluma, E. , Luke, R. , Ogbuanu, I. U. , Bassat, Q. , Kincardett, M. , Mandomando, I. , Rakislova, N. , Varo, R. , … Kotloff, K. L. (2024). Contribution of malnutrition to infant and child deaths in Sub‐Saharan Africa and South Asia. British Medical Journal Global Health, 9, e017262. 10.1136/bmjgh-2024-017262 PMC11624724 39638608

[eph70431-bib-0030] Martin, A. M. , Sun, E. W. , Rogers, G. B. , & Keating, D. J. (2019). The influence of the gut microbiome on host metabolism through the regulation of gut hormone release. Frontiers in Physiology, 10, 428. 10.3389/fphys.2019.00428 PMC6477058 31057420

[eph70431-bib-0031] Martinez, E. E. , Panciotti, C. , Pereira, L. M. , Kellogg, M. D. , Stylopoulos, N. , & Mehta, N. M. (2020). Gastrointestinal hormone profiles associated with enteral nutrition tolerance and gastric emptying in pediatric critical illness: A pilot study. Journal of Parenteral and Enteral Nutrition, 44(3), 472–480. 10.1002/jpen.1678 31304610

[eph70431-bib-0032] Martins, V. J. B. , Toledo Florêncio, T. M. M. , Grillo, L. P. , Do Carmo P Franco, M. , Martins, P. A. , Clemente, A. P. G. , Santos, C. D. L. , Vieira, M. D. E. F. A. , & Sawaya, A. L. (2011). Long‐Lasting effects of undernutrition. International Journal of Environmental Research and Public Health, 8(6), 1817–1846. 10.3390/ijerph8061817 PMC3137999 21776204

[eph70431-bib-0033] Mulenga, C. , Sviben, S. , Chandwe, K. , Amadi, B. , Kayamba, V. , Fitzpatrick, J. A. J. , Mudenda, V. , & Kelly, P. (2022). Epithelial abnormalities in the small intestine of zambian children with stunting. Frontiers in Medicine, 9, 849677. 10.3389/fmed.2022.849677 PMC8966729 35372420

[eph70431-bib-0034] Nabwera, H. M. , Bernstein, R. M. , Agbla, S. C. , Moore, S. E. , Darboe, M. K. , Colley, M. , Jallow, A. T. , Bradbury, R. , Karafin, J. , Fulford, A. J. , & Prentice, A. M. (2017). Hormonal correlates and predictors of nutritional recovery in malnourished African children. Journal of Tropical Pediatrics, 64(5), 364–372. 10.1093/tropej/fmx075 PMC6166213 29092084

[eph70431-bib-0035] Page, L. , McCain, E. , & Freemark, M. (2025). Adaptive responses in severe acute malnutrition: Endocrinology, metabolomics, mortality, and growth. Nutrients, 17(17), 2864. 10.3390/nu17172864 PMC12430813 40944251

[eph70431-bib-0036] Palacio, A. C. , Pérez‐Bravo, F. , Santos, J. L. , Schlesinger, L. , & Monckeberg, F. (2002). Leptin levels and IgF‐binding proteins in malnourished children: Effect of weight gain. Nutrition, 18(1), 17–19. 10.1016/s0899-9007(01)00690-6 11827758

[eph70431-bib-0037] Prentice, A. M. , Moore, S. E. , Collinson, A. C. , & O'Connell, M. A. (2002). Leptin and undernutrition. Nutrition Reviews, 60(10), S56–S67. 10.1301/002966402320634940 12403085

[eph70431-bib-0038] Prodam, F. , Monzani, A. , Ricotti, R. , Marolda, A. , Bellone, S. , Aimaretti, G. , Roccio, M. , & Bona, G. (2014). Systematic review of Ghrelin response to food intake in pediatric age, from neonates to adolescents. The Journal of Clinical Endocrinology & Metabolism, 99(5), 1556–1568. 10.1210/jc.2013-4010 24601727

[eph70431-bib-0039] Rehfeld, J. F. (1998). Accurate measurement of cholecystokinin in plasma. Clinical Chemistry, 44(5), 991–1001. 9590372

[eph70431-bib-0040] Smith, M. I. , Yatsunenko, T. , Manary, M. J. , Trehan, I. , Mkakosya, R. , Cheng, J. , Kau, A. L. , Rich, S. S. , Concannon, P. , Mychaleckyj, J. C. , Liu, J. , Houpt, E. , Li, J. V. , Holmes, E. , Nicholson, J. , Knights, D. , Ursell, L. K. , Knight, R. , & Gordon, J. I. (2013). Gut microbiomes of Malawian twin pairs discordant for kwashiorkor. Science, 339(6119), 548–554. 10.1126/science.1229000 PMC3667500 23363771

[eph70431-bib-0041] Soliman, A. T. , ElZalabany, M. M. , Salama, M. , & Ansari, B. M. (2000). Serum leptin concentrations during severe protein‐energy malnutrition: Correlation with growth parameters and endocrine function. Metabolism, 49(7), 819–825. 10.1053/meta.2000.6745 10909989

[eph70431-bib-0042] Sturgeon, J. P. , Mutasa, K. , Bwakura‐Dangarembizi, M. , Amadi, B. , Ngosa, D. , Dzikiti, A. , Chandwe, K. , Besa, E. , Mutasa, B. , Murch, S. H. , Hill, S. , Playford, R. J. , VanBuskirk, K. , Kelly, P. , & Prendergast, A. J. (2025). Therapeutic interventions targeting enteropathy in severe acute malnutrition modulate systemic and vascular inflammation and epithelial regeneration. EBioMedicine, 111, 105478. 10.1016/j.ebiom.2024.105478 PMC11697704 39662176

[eph70431-bib-0043] Sturgeon, J. P. , Njunge, J. M. , Bourke, C. D. , Gonzales, G. B. , Robertson, R. C. , Bwakura‐Dangarembizi, M. , Berkley, J. A. , Kelly, P. , & Prendergast, A. J. (2023). Inflammation: The driver of poor outcomes among children with severe acute malnutrition? Nutrition Reviews, 81(12), 1636–1652. 10.1093/nutrit/nuad030 PMC10639108 36977352

[eph70431-bib-0044] Sung, Y. , Yu, Y. C. , & Han, J. M. (2023). Nutrient sensors and their crosstalk. Experimental and Molecular Medicine, 55, 1076–1089. 10.1038/s12276-023-01006-z PMC10318010 37258576

[eph70431-bib-0045] The Childhood Acute Illness and Nutrition (CHAIN) Network . (2022). Childhood mortality during and after acute illness in Africa and South Asia: A prospective cohort study. Lancet Global Health, 12(10), e673–e684. 10.1016/S2214-109X(22)00118-8 PMC9023747 35427524

[eph70431-bib-0046] Vinay, E. S. , Laxmi Narsimha Rao, B. , Saf, N. , & Gautam, D. (2026). C‐peptide in precision diabetes care and beyond: A comprehensive review. Clinical Medicine Insights: Endocrinology and Diabetes, 13(19), 11795514251397811. 10.1177/11795514251397811 PMC12905110 41694179

[eph70431-bib-0047] Watkins, J. D. , Carter, S. , Atkinson, G. , Koumanov, F. , Betts, J. A. , Holst, J. J. , & Gonzalez, J. T. (2023). Glucagon‐like peptide‐1 secretion in people with versus without type 2 diabetes: A systematic review and meta‐analysis of cross‐sectional studies. Metabolism, 140, 155375. 10.1016/j.metabol.2022.155375 36502882

[eph70431-bib-0048] World Health Organization . (2023). WHO guideline on the prevention and management of wasting and nutritional oedema (acute malnutrition) in infants and children under 5years. 38498638

[eph70431-bib-0049] Wortha, S. M. , Wüsten, K. A. , Witte, V. A. , Bössel, N. , Keßler, W. , Vogelgesang, A. , & Flöel, A. (2021). Gastrointestinal hormones in healthy adults: Reliability of repeated assessments and interrelations with eating habits and physical activity. Nutrients, 13(11), 3809. 10.3390/nu13113809 PMC8624073 34836065

[eph70431-bib-0050] Zambia Statistics Agency, Ministry of Health (MoH) [Zambia], & ICF . (2024). Zambia demographic and health survey 2024: Key indicators report. Zambia Statistics Agency, MoH, and ICF.

